# No Association of Xenotropic Murine Leukemia Virus-Related Viruses with Prostate Cancer

**DOI:** 10.1371/journal.pone.0019065

**Published:** 2011-05-04

**Authors:** William M. Switzer, Hongwei Jia, HaoQiang Zheng, Shaohua Tang, Walid Heneine

**Affiliations:** Laboratory Branch, Division of HIV/AIDS Prevention, National Center for HIV/AIDS, Viral Hepatitis, STD, and TB Prevention, Centers for Disease Control and Prevention, Atlanta, Georgia, United States of America; Virginia Commonwealth University, United States of America

## Abstract

**Background:**

The association of the xenotropic murine leukemia virus-related virus (XMRV) with prostate cancer continues to receive heightened attention as studies report discrepant XMRV prevalences ranging from zero up to 23%. It is unclear if differences in the diagnostic testing, disease severity, geography, or other factors account for the discordant results. We report here the prevalence of XMRV in a population with well-defined prostate cancers and RNase L polymorphism. We used broadly reactive PCR and Western blot (WB) assays to detect infection with XMRV and related murine leukemia viruses (MLV).

**Methodology/Principal Findings:**

We studied specimens from 162 US patients diagnosed with prostate cancer with a intermediate to advanced stage (Gleason Scores of 5–10; moderate (46%) poorly differentiated tumors (54%)). Prostate tissue DNA was tested by PCR assays that detect XMRV and MLV variants. To exclude contamination with mouse DNA, we also designed and used a mouse-specific DNA PCR test. Detailed phylogenetic analysis was used to infer evolutionary relationships. RNase L typing showed that 9.3% were homozygous (QQ) for the R462Q RNase L mutation, while 45.6% and 45.1% were homozygous or heterozygous, respectively. Serologic testing was performed by a WB test. Three of 162 (1.9%) prostate tissue DNA were PCR-positive for XMRV and had undetectable mouse DNA. None was homozygous for the QQ mutation. Plasma from all three persons was negative for viral RNA by RT-PCR. All 162 patients were WB negative. Phylogenetic analysis inferred a distinct XMRV.

**Conclusions and Their Significance:**

We found a very low prevalence of XMRV in prostate cancer patients. Infection was confirmed by phylogenetic analysis and absence of contaminating mouse DNA. The finding of undetectable antibodies and viremia in all three patients may reflect latent infection. Our results do not support an association of XMRV or MLV variants with prostate cancer.

## Introduction

Prostate cancer is one of the most frequent, slow growing, noncutaneous malignancies of men in the developing world [1]. For example, it is estimated that over 192,000 men in the US, mostly of African-American descent, will be diagnosed with prostate cancer this year [2], [3]. Although the natural history of prostate cancer is currently unknown, multiple etiologies have been hypothesized, including genetic defects [4], [5], [6]. In 2006, using microarray and RT-PCR analysis xenotropic murine leukemia virus (MLV)-related virus (XMRV) was first identified in about 40% of familial prostate cancer patients containing the R462Q mutation in the RNase L gene, a component of the antiviral innate immunity [7]. MLVs are endogenous gammaretroviruses that have integrated into the mouse genome and can cause cancer, neurologic disease, and immunodeficiency disorders in mice and are classified into three groups based on their host tropism [8]. Xenotropic MLV (XMLV) replicate only in non-mouse cells. In contrast, ecotropic MLV (EMLV) replicate only in mice, while polytropic MLV (PMLV) have a broader tropism and can replicate in mouse and non-mouse hosts [8]. XMRV shares about 93% and 89% nucleotide identity with XMLV and PMLV across the genome [7]. Identification of a possible viral cause of prostate cancer is highly significant because it could facilitate treatment and prevention of this debilitating disease.

Additional evidence for XMRV infection of persons with prostate cancer has been reported in a US study showing a higher prevalence of XMRV DNA detection by PCR (6%) and viral proteins by immunohistochemistry (IHC) (23%) in prostate tissues from 334 prostate cancer patients compared to 101 benign controls (1.9% by PCR and 3.9% by IHC) [9]. This study also reported finding XMRV more frequently in patients with a higher prostate tumor grade suggesting a causal link between virus and disease. A similar trend was reported in another study that reported a 22% XMRV prevalence rate in prostate cancer patients from Texas but found virus in both tumor and non-tumor tissues [9], [10]. XMRV neutralizing antibodies were identified recently in 11 of 40 (27.5%) US prostate cancer patients and XMRV sequences were confirmed in five of these 11 persons by using nested DNA PCR and fluorescence in situ hybridization (FISH) [11].

In contrast, studies in Europe and two from the US reported little or no XMRV infection [12], [13], [14], [15], [16]. One study of German prostate cancer patients and one in The Netherlands found a much lower XMRV prevalence (1/87, 1.2% and 3/74 = 4%, respectively) using only RT-PCR and XMRV-specific primers [12]. A second larger study in Germany using a combination of DNA and RNA PCR found no evidence of XMRV infection in 589 patients [14]. In addition, sera from an additional 146 prostate cancer patients were all negative by testing with an ELISA incorporating recombinant XMRV envelope (Env) and Gag proteins and by indirect immunofluorescence assays expressing XMRV [14]. XMRV was also not found in DNA from tissues from 161 and 200 prostate cancer patients from two U.S. populations, respectively [13], [15]. The study by Aloia *et al.* also did not detect XMRV proteins in tissues from 596 prostatic adenocarcinomas and 452 benign prostate tissue specimens using IHC [13].

The reasons for the incongruent XMRV results are not known but may be related to technical differences in XMRV testing or to factors related to the patient populations, including disease stage, genetic factors, or geographical clustering. Similar discordant results have also been reported recently for XMRV in persons with the chronic fatigue syndrome (CFS) [17], [18], [19], [20], [21], [22], [23], including a study by Lo *et. al* reporting PMLV-like sequences among US patients with CFS [24]. The majority of the prostate cancer studies utilized PCR with XMRV-specific primers which may not be highly sensitive for other MLV-like variants [7], [9], [10], [11], [12], [15], [16]. In addition, most studies did not perform serologic testing for antibodies that are hallmarks of retroviral infections and can, thus, provide additional evidence of infection that is not tissue-specific.

We report here XMRV testing of a well-characterized cohort of 162 prostate cancer patients from the US by using a combination of serologic and PCR assays capable of detecting XMRV and PMLV. We also measured the distribution of the RNase L QQ mutation to evaluate the association of XMRV with this mutant allele [7]. We also have used a sensitive PCR test to detect contamination with murine DNA and exclude false positive PCR results. Our findings show that XMRV is rare in this patient population and do not support an association of this virus with prostate cancer.

## Results

### Rare XMRV sequences in tumor tissues from prostate cancer patients

**Figure 1:**
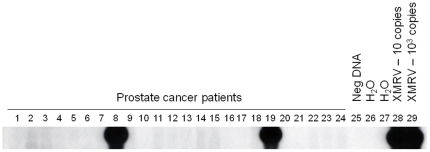
Identification of XMRV sequences in prostate cancer patients. Representative nested *pol* PCR results using prostate tumor DNA. Lanes 1–24, prostate cancer patients, including patients 5956 (lane 8) and 6203 (lane 19); lane 25, negative human PBMC DNA control; lanes 26 and 27, water only controls for primary and nested PCR tests, respectively; lanes 28 and 29, assay sensitivity controls consisting of 10 and 10^3^ copies of XMRV VP62 plasmid DNA diluted in a background of 1 ug of human PBMC DNA, respectively.

ß-actin sequences were detected by PCR in the DNA extracts from all prostate tissues confirming their integrity for PCR testing. DNA from only two (5956 and 6203) of 162 patients (1.23%) tested positive for XMRV sequences in the initial screening (Fig. 1, Table 1). Of the 77 DNA specimens that were additionally tested in triplicate by the *pol* PCR assay, only one patient (5935) (1.3%) was found positive. This specimen was positive in only one of three replicates. Thus, the overall PCR prevalence was 3/162 or 1.85%. Patient 5956 was positive for *gag* and *pol* sequences in 3/5 and 4/6 repeat tests, respectively, and *env* sequences were also detected in 1/3 repeat tests (Table 2). Specimen 5956 was also positive in all three replicate *pol* PCR tests. Only XMRV *pol* and *env* sequences were detected in patient 6203 prostate tissue DNA in 1/4 and 1/1 repeat tests, respectively. Additional testing of DNA extracted from another prostate tissue section of 6203 was repeatedly negative, likely reflecting an uneven distribution of low copy XMRV in this tissue. DNA from all three patients repeatedly tested negative for murine mtDNA demonstrating the absence of contaminating mouse DNAs. To test for viremia we analyzed RNA extracts from plasma specimens from all three patients. All samples had undetectable sequences by the qRT-PCR and nRT-PCR tests.

**Table 1:** XMRV/MLV infection is rare in prostate cancer^1^^,^ 2.

		RNase L R462Q Genotype			XMRV/MLV nPCR^3^		Serology
Sample Type	Sample Total	RR	RQ	QQ	GAG	POL	WB
ProstateTissue DNA	162	74 (45.7)	73 (45.1)	15 (9.3)	1 (0.6)	3 (1.9)	-
Plasma	162	-	-	-	-	-	0/162 (0)

1 Percentages in parentheses.

2 Dashes indicate test not performed on these sample types.

3 nPCR, nested PCR.

**Table 2:** Detection of XMRV in three prostate cancer patients^1^.

			XMRV/MLV nPCR^3^			qRT-PCR^4^	nRT-PCR^5^	Murine PCR^6^
Patient ID	SpecimenType^2^	RNase L Q462R genotype	*gag*	*pol* 7	*env*	*pro*	*gag*	MCOX2
5935	PTT DNA	RQ	0/3	1/4	0/3	-	-	0/1
	Plasma		-	-	-	0/1	0/3	-
5956	PTT DNA	RR	3/5	7/9	1/3	-	-	0/3
	Plasma	-	-	-	-	0/2	0/3	-
6203	PTT DNA	RQ	0/4	1/4	1/1	-	-	0/2
	Plasma	-	-		-	0/2	0/3	-

1 Dashes indicate test not performed.

2 PTT, prostate tumor tissue.

3 nPCR, nested PCR; *gag*, group specific antigen; *pol*, polymerase; *env*, envelope.

4 qRT-PCR, quantitative RNA PCR; *pro*, protease gene.

5 nRT-PCR, nested RNA PCR.

6 Murine PCR, test for detection of specimen contamination with mouse cells or DNA using mitochondrial primers (MCOX2).

7 For specimens 5935 and 5956 testing includes results of triplicates and the initial screening. Quantity of DNA for 6203 was insufficient for triplicate testing.

### Identification of XMRV diversity in prostate cancer patients

Sequence analysis showed that patients 5935, 5956 and 6203 are infected with variant XMRV strains. The 168-bp *pol* sequences from all three patients showed 90.5–100% nucleotide identity to each other, 94–100% to XMRV, 91.7–98.8% to XMLV, 94–100% to PMLV, and 91–100% to ecotropic MLV (EMLV) in this short region. 164-bp *env* sequences from persons 5956 and 6203 were identical to each other and shared the highest nucleotide identity (94.9–100%) to XMRV and other xenotropic MLV strains, respectively, [25]. The *env* sequences from both persons were highly divergent from EMLVs sharing only about 46% nucleotide identity. 413-bp *gag* sequences were only amplified from the prostate tissue DNA of person 5956 and had about 98% nucleotide identity to XMRV but was identical to a xenotropic mERV found on mouse chromosome 8 (GenBank accession # AC127575).

**Figure 2:**
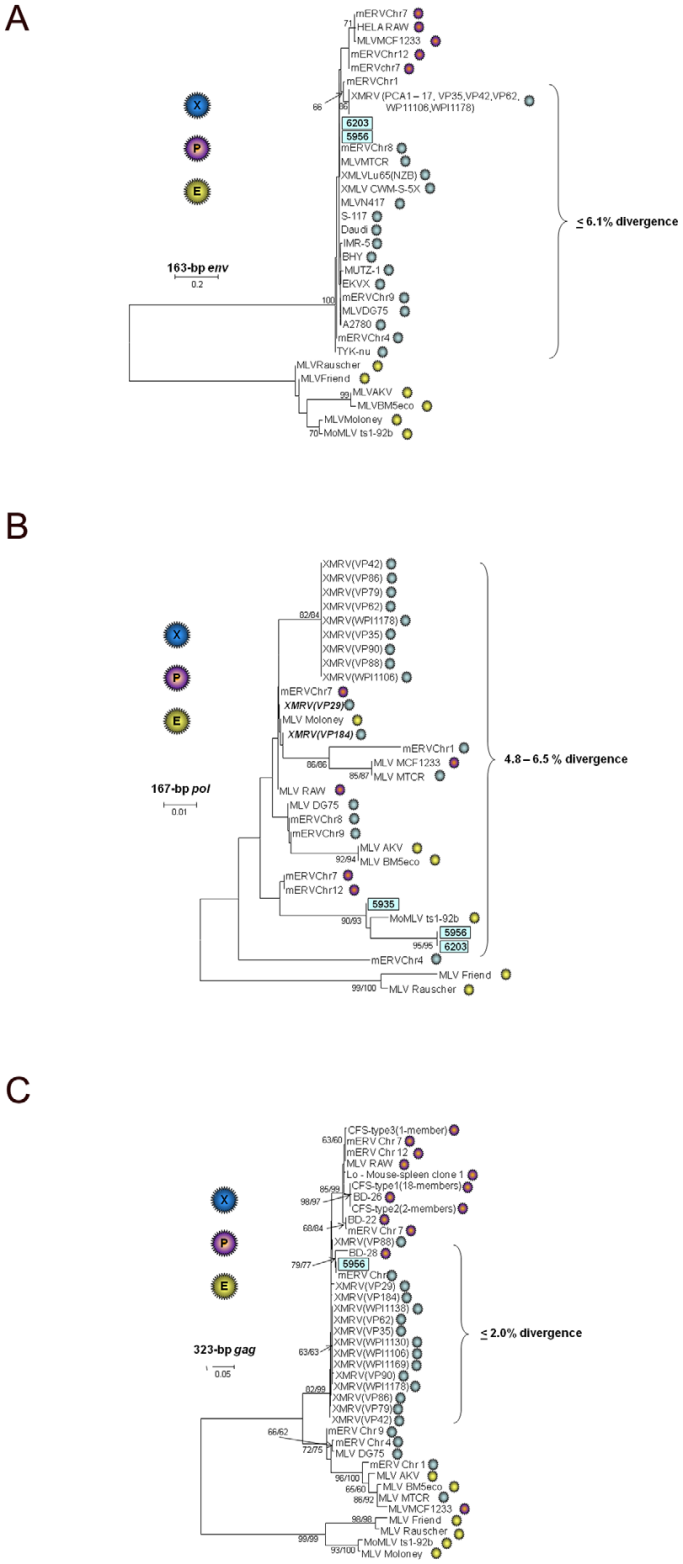
Identification of variant XMRV in prostate cancer patients using phylogenetic analysis. A. envelope (*env*), B. polymerase (*pol*), and C. *gag*. Stability of the tree topology was tested using 1000 bootstrap replicates in both neighbor joining (NJ) and maximum likelihood (ML) methods. Bootstrap values >60 are shown at major nodes (NJ/ML). New sequences from the current study are boxed. Accession numbers for prototypical MLV sequences available at GenBank are XMRV VP35 = DQ241301, XMRV VP62 = DQ399707, XMRV VP42 = DQ241302, XMRV WPI-1106 = GQ497344, XMRV WPI-1178 = GC497343, XMRV PCA1–PCA17 = GU812341–GU812357, MLV DG-75 = AF221065, MLV MTCR = NC_001702, MLV AKV = J01998, MLV BM5eco = AY252102.1, Moloney MLV = J02255, Moloney neuropthogenic MLV variant ts1-92b = AF462057, Rauscher MLV = NC_001819, Friend MLV = X02794, mERV Chr 7 = AC167978, mERV Chr 7 = AC127565, mERV Chr 8 = AC127575, mERV Chr 12 = AC153658, mERV Chr 9 = AC121813, mERV Chr 4 = AL627077, mERV Chr 1 = AC083892), XMLV A2780 = FR670594, XMLV BHY = FR670595, XMLV Daudi = FR670596, XMLV EKVX = FR670597, XMLV IMR-5 = FR670598, XMLV MUTZ-1 = FR670599, XMLV S-117 = FR670600, XMLV TYK-nu = FR670601. Sequences denoted RAW are from the polytropic MLV isolated in HeLa cells used to develop the in-house WB test. Sequences coded as XMRV VP and PCA and WPI are from prostate cancer and CFS patients, respectively. Additional prostate cancer patient VP *gag* and *pol* sequences were kindly provided by Drs. Robert Silverman and Joe Derisi. Viral tropism, as determined by analysis of *env* sequences, is indicated by blue (xenotropic), purple (polytropic), and yellow (ecotropic) spheres.

Phylogenetic analysis of the *env* sequences inferred three distinct clusters with strong bootstrap support based on viral tropism as expected, since this region includes a portion of the viral surface membrane involved in cellular tropism (Fig. 2a). The *env* sequences from both patients clustered with XMLV sequences but were distinct from previously reported XMRV and prototypical PMLV (Fig. 2a). In contrast, the inferred phylogenetic relationships of *pol* sequences from all MLV groups showed that this region does not cluster by host range (Fig. 2b). The three *pol* sequences from the prostate cancer patients formed a well-supported lineage containing a neuropathogenic Moloney MLV recombinant (strain ts-1-92b, GenBank accession # AF462057) (Fig. 2b). XMRV *pol* sequences from previously reported prostate cancer patients (coded with VP), except two (VP29 and VP184, kindly provided by Joe DeRisi), clustered with those from persons with CFS (coded with WPI) with significant bootstrap support. VP29 and VP184 *pol* sequences were identical to each other but 1.2% divergent from other XMRV and formed a weakly supported cluster containing a mixture of EMLV and PMLV (Fig. 2b). These results demonstrate a broader diversity of XMRV in this region than previously seen, but are consistent with recombination with Moloney MLV as reported recently [7], [25]. VP29, VP79, VP86, VP88, VP90, and VP184 are prostate cancer patients reported in the Urismann *et al.* paper but for which the XMRV *pol* sequences are not yet available at GenBank [7]. Newly reported *gag* and *pol* sequences found in human tumor cell line DNA were located outside the regions used in our study and thus were not included in the analyses [25].

The *gag* sequence from patient 5956 clustered with XMRV sequences from a prostate cancer patient (VP88), a xenotropic mERV found on mouse chromosome 8, and a PMLV sequence identified in a blood donor (BD-28) by Lo *et al.* (Fig. 2c) [24]. This lineage was between clades containing XMRV from prostate cancer and CFS patients and most PMLVs and the remaining MLVs found in the Lo *et al.* study [24]. One prototypical PMLV (MCF1233) clustered with all EMLVs and two XMRVs, suggesting it is a recombinant in this region (Fig. 2c).

These phylogenetic results also suggest the short region of *env* targeted by our new PCR assay is useful for determining viral tropism for typing the MLV-related sequences. This information may be helpful in understanding the origin of the sequences identified in PCR-positive humans. In contrast, the *gag* and *pol* regions showed less clustering by tropism indicating viral recombination in these regions. For example, the *gag* sequences of prototypical XMLVs (MTCR), EMLVs (AKV, BM5eco), and PMLVs (MCF1233) clustered together with highly significant bootstrap support (Fig. 2a).

Nearly identical phylogenetic tree topologies for each gene region were obtained with both the NJ and ML methods. The XMRV sequences from both prostate cancer patients were also distinct from the PMLV sequences amplified from the murine cell line (RAW) used for preparing WB antigens demonstrating further that these are not laboratory contaminants (Fig. 2). These results confirm the presence of XMRV in both patients and demonstrate that XMRV diversity is greater than currently appreciated.

The XMRV sequences presented in the current paper are available at GenBank with the accession numbers HM003608–HM003612, and HQ116790. Sequences from other studies with XMRV-positive prostate cancer patients were not available at GenBank for inclusion in our analyses [9], [11], [12], [16].

### Absence of antibodies in plasma from prostate cancer patients

**Figure 3:**
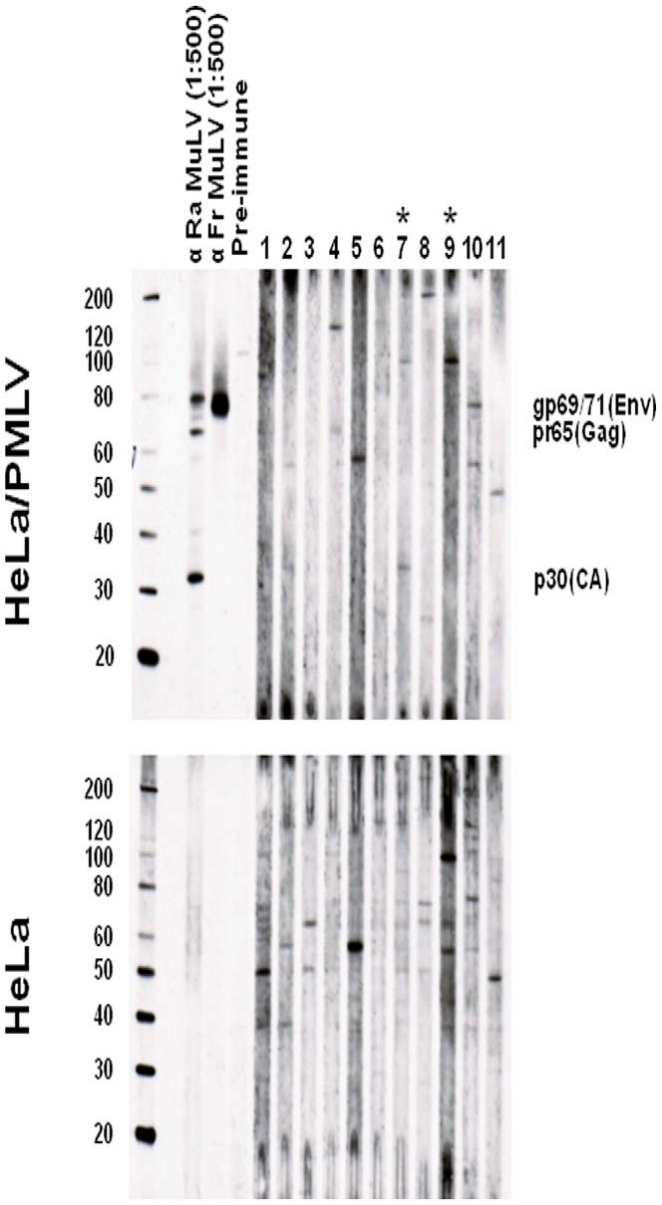
Absence of antibodies to XMRV and MLV in prostate cancer patients. Molecular weight markers (kD) are provided on the left of the WBs in the upper panels. Expected sizes of viral Gag (p30, capsid (CA)), pr65, and Envelope (Env, gp69/71) proteins are provided in each WB in the upper panels. Representative WB results for eleven prostate cancer patients, including patients 5956 and 6203 (indicated with asterisks). Determination of MLV specific reactivity is determined by comparison of seroreactivity to xenotropic MLV-infected HeLa antigens and uninfected HeLa antigens in upper and lower panels, respectively. Ra, Rauscher MLV whole virus goat polyclonal antisera; Fr, Friend MLV Envelope (gp69/71) goat polyclonal antisera; pre-immune, goat sera prior to immunization.

All 162 prostate cancer patient plasma samples were found to be negative for antibodies to XMRV/MLV in the WB test, including plasma from persons 5935, 5956, and 6203 (Fig. 3). In most plasma specimens some level of nonspecific reactivity was observed to the uninfected antigen but without evidence of specific reactivity to Gag and/or Env proteins in the infected antigen blot (Fig. 3).

### RNase L R462Q allelic distribution in the study population and clinical characteristics of the three XMRV-infected persons

15 of the 162 persons (9.3%) were determined to be homozygous (QQ) for the R462Q RNase L mutation, 74 (45.6%) had the homozygous RR allele, and 73 (45.1%) were heterozygous (RQ) for the mutation (Table 1).

Patient 5935 is the heterozygous RQ RNase L genotype. He has a poorly differentiated adenocarcinoma with a Gleason's score of 6, T2C pathologic stage, PSA level of 4.6 ng/mL. Patient 5956 is the wild-type homozygous RR RNase L genotype. He had a poorly differentiated adenocarcinoma with a Gleason's score of 7, T3A pathologic stage, a 12.4 ng/mL PSA level. Patient 6203 is the heterozygous RQ RNase L genotype, and had a moderately differentiated adenocarcinoma with a Gleason's score of 6, T2C pathologic stage, and a 7.1 ng/mL PSA level. Thus, we were unable to confirm an association of XMRV infection with the homozygous QQ RNase L allele. Given the low XMRV prevalence, we did not have the statistical power to determine if there is an association of XMRV with tumor grade.

## Discussion

We used PCR and serologic testing to study the prevalence of XMRV in 162 well-characterized North American patients with prostate cancer. We surveyed cancers with intermediate to advanced stages, and utilized multiple PCR assays, including tests with conserved primers to allow the detection of diverse XMRV and MLV-related sequences. Nearly half the specimens were also tested in triplicate to maximize the detection sensitivity of low-copy sequences. We used a broadly reactive WB assay to test all patients for antibodies. Our findings document a very low prevalence (1.9%) of XMRV sequences in prostate tissue DNA and absence of antibody positivity in all specimens. Combined these data do not support an association of XMRV or related MLV with prostate cancer.

The predominantly negative PCR results were observed despite the use of 1 ug of DNA which is 4–10× higher than the input DNA used in previous studies that reported XMRV detection [7], [9], [14], [16]. Likewise, the observed low prevalence of XMRV may not be explained by a decreased PCR assay sensitivity since we screened specimens with the same assay used originally by Urisman *et al.* [7]. In the three specimens with detectable sequences, we noted that the copy number was very low as nested PCR and replicate testing was often needed for detection. Importantly, in all three specimens we were unable to detect mouse mtDNA despite the use of a highly sensitive assay, which suggests that the source of XMRV is unlikely the result of contamination with mouse DNA. These results are important since mouse DNA contamination was reported to be the source of XMRV in a recent study of prostate cancer tissues [26]. The authors of this study used a PCR assay for the mouse intracisternal A particle (IAP) which they reported to be more sensitive than a mouse-specific D-loop mtDNA-based assay [26]. Although DNA specimens were not available for testing in the IAP test, we have found that the IAP assay is equally sensitive to our COX2 real-time mtDNA assay (data not shown), thus further excluding mouse DNA contamination as a source for our positive PCR results. In addition, the DNA specimens were prepared at FCCC where only human biological specimens, and not cell lines, are handled, reducing further the possibility of murine contamination.

Sequence analysis of the PCR-positive specimens was highly informative because it confirmed that all three specimens were XMRV-related. Also, the finding of a viral strain in three prostate cancer patients that is distinct from the XMRV seen in previous studies is significant and demonstrates a broader viral diversity [7], [9], [14], [16]. This would be an expected result consistent with virus evolution during spread and persistence. The absence of antibodies and plasma viremia in these three patients is noteworthy because it may reflect sequestered or latent infections. Loss of antibody during a latent infection, while atypical of most retroviral infections, has been described previously for natural infection of macaques with simian type D retroviruses (SRV) [27]. SRV in macaques is associated with outbreaks of severe immune deficiency in primate colonies and latent SRV infection in these antibody negative animals is confirmed with greater sensitivity using PCR analysis [27]. Our results are also consistent with those seen recently in macaques experimentally infected with XMRV in which tissues at necropsy are PCR-positive but viremia and detection of provirus in PBMCs disappear quickly, followed by loss of antibody detection [28], [29]. Although one study reported the detection of XMRV neutralizing antibody in 11 patients with prostate cancer, these data were not confirmed by more sensitive methods such as WB, or by PCR testing in all cases, and thus may represent nonspecific reactivity [11], [17]. Longitudinal studies may better define host responses to XMRV infection.

Our study population contained 15 persons with the mutant QQ RNase L allele. However, none were XMRV-positive, which contrasts with the original findings by Urismann *et al.* [7]. The XMRV-infected persons in our study had either the homozygous wild-type (RR) or heterozygous (RQ) RNase L R462Q alleles. Our results are consistent with other US and European studies which also identified higher frequencies of the homozygous QQ mutant allele in XMRV-negative persons [9], [10], [16]. Combined, these results suggest that XMRV infection is not associated with this allelic form of RNase L.

Although our data do not support an association of prostate cancer with XMRV, it is important to understand whether XMRV has any causal role in prostate cancer when it is infrequently detected. In general, gammaretroviruses like XMRV induce malignant transformation by insertional mutagenesis, so that malignant cells in a tumor are all clonally infected. This mechanism of carcinogenesis has been found in animals as well as in children exposed to MLV-derived vectors in gene-therapy trials [30], [31]. Therefore, the low-frequency of XMRV-infected cells found in all three patients are inconsistent with a direct role of XMRV in the prostate carcinogenesis in these patients. Previously, a human prostate cancer cell line, 22Rv1, has been shown to express high levels of XMRV, a finding that supports a role of XMRV in prostate cancer tumorigenesis [32]. However, it was reported recently that the XMRV expressed by 22Rv1 may not be of human origin, but most likely arose via recombination of two overlapping XMRV-like genomes during passage of the prostate tumor in inbred mice (T. Paprotka *et al.* 18^th^ Conference on Retroviruses and Opportunistic Infections). Furthermore, others have shown that XMRV integration sites cloned from prostate tissues of two of nine patients may have been the result of contamination with DNA from experimentally infected DU145 cells [33], while other XMRV integration sites are not near tumor suppressor genes or proto-oncogenes [34]. Combined, these results raise questions about the role of XRMV in prostate cancer. Nonetheless, more work is needed to better understand the prevalence of XMRV and MLVs in humans and their role in human disease.

## Materials and Methods

### Study population

Anonymous, archived plasma and prostate tissue were available from 162 U.S. prostate cancer patients collected by the Fox Chase Cancer Center (FCCC) Biosample Repository Core Facility between 1997 and 2004. Whole blood specimens were collected at the time of pre-surgical testing or on the day of surgery prior to anesthesia from consenting cancer patients as part of a study approved by the FCCC Human Subjects Committee. Written informed consent was obtained from all participants. The FCCC Human Subjects Ethics Committee approved collection, storage, and future testing of the specimens collected from all consenting study participants. A non-research determination was approved for testing of the anonymized samples at CDC for XMRV and related viruses. The age at diagnosis of the 162 participants ranged from 36 to 71 years old with an average and median of 57 years. 86% were Caucasian, 12% African American, and 1% were Asian, not recorded, or other. The average and median serum prostate-specific antigen (PSA) levels were 7.22 ng/mL and 5.5 ng/mL, respectively. The tumor grade for 75 of 162 (46%) of the participants was moderately differentiated, while 54% had poorly differentiated tumors. Using the Gleason system, 42% of the study population had grade 5–6 cancer, 40% had grade 7, and 18% had high grade adenocarcinoma (Gleason's score 8–10). Pathologic staging classified the prostate tumors as T1C (0.6%), T2A (9.3%), T2B (3.1%), T2C (61.7%), T3A (17.9%), T3B (5.6%), and T4 (1.8%).

### Specimen preparation

Plasma was aliquoted and stored at −80°C within 4 hours of blood collection. Plasma samples were aliquoted again at the CDC after thawing for serologic testing; the remaining aliquots were frozen at −80°C. DNA samples were prepared by phenol-chloroform extraction of prostate tissues at FCCC using standard procedures. Only human tissues are processed at FCCC. For selected samples the QIAamp DNA minikit (Qiagen, Carlsbad, CA) was used at the CDC. For plasma RT-PCR testing a volume of 0.5–1.0 ml was ultracentrifuged at 45,000 rpm to concentrate virus. 360 ul to 860 ul plasma supernatant was carefully removed and the pellet was resuspended in 140 ul of centrifuged plasma remaining in the tube and then RNA was extracted using the QIAamp Viral RNA minikit (Qiagen, Valencia, CA). Nucleic acid concentrations were determined by spectrophotometry using the Nanodrop instrument (Thermo Scientific, Wilmington, DE). Integrity of the DNA and RNA specimens was determined using ß-actin or GAPDH PCR, respectively, as described [35], [36]. All specimen preparation, tissue culture, and PCR testing was done in physically isolated rooms to prevent contamination.

### Molecular detection of XMRV

To detect XMRV and other MLV variants we used separate PCR assays with primers specific for the detection of XMRV, or conserved primers for the generic detection of MLV and XMRV, as previously described [21]. DNA specimens were screened using two nested PCR assays. The first assay uses the PCR primers GAGOF, GAGOR, GAGIF, and GAGIR employed by Urismann *et al.* and by Lombardi *et al.* to detect 413-bp XMRV *gag* sequences in prostate cancer patients and in persons with CFS, respectively [7], [18]. The second PCR assay generically detects MLV and XMRV polymerase (*pol*) sequences about 216-bp in length using the primers XPOLOF, XPOLOR, XPOLIF, and XPOLOR [21]. Both the *gag* and *pol* PCR tests can detect 10 copies of XMRV plasmid DNA diluted in a background of 1 ug of human DNA [21]. Specimens testing positive for either *gag* or *pol* sequences were re-tested with both assays and were also tested with a third nested PCR test for XMRV envelope (*env*) sequences that is also generic for XMRV/MLV. The external XENVOF (5′ GGG GAT CTT GGT GAG GGC AGG AGC 3′) and XENVOR (5′ CAG AGA GAA CAG GGT CAC CGG GTC 3′) and internal primers XENVIF (5′ AGG GCT ACT GTG GCA AAT GGG GAT 3′) and XENVIR (5′ CCT TTT ACC CGC GTC AGT GAA TTC 3′) amplify a 215-bp *env* sequence. In addition, a subset of specimens (n = 77) for which sufficient DNA was available were tested in triplicate using the nested *pol* PCR assay to improve detection of low copy XMRV/MLV.

PCR was performed using 1 ug of prostate tissue DNA in a 100 ul reaction volume using standard conditions of 94°C for 30 sec, 50°C for 30 sec, and 72°C for 45 sec for 40 cycles on an ABI 9700 thermalcycler (Foster City, CA). PCR products were visualized by electrophoresis in an ethidium bromide-stained 1.8% agarose gel. To increase the sensitivity and specificity of the assays, amplified *gag*, *pol*, and *env* sequences were confirmed by Southern blot analysis using the biotinylated oligoprobes XGAGP2 (5′ ACC TTG CAG CAC TGG GGA GAT GTC 3′), XPOLP (5′ TTG ATG AGG CAC TGC ACA GAG ACC 3′), and XENVP (5′ TGG GCT CCG GTA GCA TCC AGG GTG 3′) and chemiluminescence detection. Like the XMRV *gag* and *pol* PCR assays [21], the new XMRV *env* PCR test was also capable of detecting 10 copies of XMRV plasmid in a background of 1 ug human DNA (30/32, 93.8%). We did not detect any XMRV/MLV sequences in PBMC DNA from 41 US blood donors using each of the three nested PCR tests [21].

### Quantitative XMRV RNA RT-PCR

Plasma samples from persons with positive XMRV PCR results in the prostate tissue DNA were tested further for viral RNA using two RT-PCR tests. The first assay, referred to as q*pro* uses MLV/XMRV generic protease (*pro*) Taqman primers Pro-UNV-F1 (5′ CCT GAA CCC AGG ATA ACC CT 3′) and Pro-UNV-R1 (5′ GTG GTC CAG CGA TAC CGC T 3′) and probes Pro-UNV-P1C (FAM5′ AGA TAC TGG GGC CCA ACA CTC CGT GCT GAC 3′BHQ1) and Pro-UNV-PR1 (FAM5′ CCT CCA GTA GCC CCT TGG ACC CAG GC 3′BHQ1). The second assay is the nested GAG RT-PCR test used by Urismann *et al.* and Lombardi *et al.* using the primers GAGOF, GAGOR, GAGIF, and GAGIR to detect XMRV RNA sequences in prostate cancer patients and persons with CFS [7], [18], respectively. For the Taqman RT-PCR test, 10 ul of RNA extracted from 50 ul plasma equivalents, 900 nM of primers and 250 nM of probes were used in a one step RT-PCR reaction mixture at 45°C for 20 min, 95°C for 10 min, followed by 55 cycles of 95°C for 30 sec, 52°C for 30 sec and 62°C for 30 sec. A plasmid containing the full-length genome of XMRV (VP62) and RNA extracted from tissue culture supernatants from the XMRV(VP62)-infected DU145 cell line (C7) (both kindly provided by Robert Silverman) were used as positive controls for the assay and for generating the standard curve for viral quantification [7]. The qRT-PCR *pro* assay has a sensitivity of 10 copies of XMRV protease RNA sequences per reaction in replicate dilutions (40/40, 100%) and a linear range from 10^1^ to 10^8^ copies. Plasma from 32 US human blood donors and 32 HIV-1-infected persons tested negative by this assay.

For the nested *gag* RT-PCR tests, cDNA was synthesized with the XMRV-specific reverse primer GAG OR by using iScript Select cDNA Synthesis Kit (Bio-Rad, Hercules, CA) in a 20 ul reaction following the manufacturer's instructions. Briefly, 10 ul RNA representing extract from 126 ul plasma was mixed with 10 uM GAGOR, 2 ul GSP enhancer solution and Nuclease-free water in a 15 ul reaction volume. Following incubation at 65°C for 5 minutes and cooling to 4°C, 4 ul of 5× iScript Select reaction mix and 1 ul reverse transcriptase were added and incubated at 42°C for 1 hr. The reaction was stopped by heating to 85°C for 5 minutes. For first round PCR, 10 ul of the RT product was mixed with 0.2 uM of the external primers GAG OF and GAG OR, 2.5 mM of each dNTP, 2.5 mM MgCl2 using the Expand High Fidelity PCR System (Roche, Indianapolis, IN) and 5 ul 10× PCR buffer in a 50 ul reaction volume for 35 cycles at 94°C for 30 sec, 52°C for 30 sec, and 72°C for 45 sec. For nested PCR, 0.2 uM of the internal primers GAG IF and GAG IR in first round PCR reaction and run for 40 cycles with annealing temperature increased to 54°C. Both primary and nested PCR products were electrophoresed on 1.8% agarose gels and specific amplicons were detected by Southern blot as previously described. The nested gag RT-PCR assay had a sensitivity between 25 and 100 copies per reaction in replicate testing of XMRV RNA 92.5% (37/40) and 97.5% (39/40), respectively, but could also detect 5 copies in 50% (20/40) of the replicates. Plasma from 40 HIV-1 infected persons were all negative by this assay. 32 of these samples were negative by the *pro* qRT-PCR test.

### Quantitative PCR for murine mitochondrial DNA (mtDNA) cytochrome oxidase subunit 2 (COX2) sequences

Contamination of specimens with mouse DNA will produce a false positive PCR result since mouse DNA contains endogenous MLV sequences that amplify by the diagnostic XMRV/MLV PCR assays. Thus, to distinguish true infection from specimen contamination with murine DNA, all specimens testing positive for XMRV/MLV sequences were also tested for murine mtDNA COX2 sequences using a newly developed PCR assay. The primers MCOX2F2 (5′ TTC TAC CAG CTG TAA TCC TTA 3′) and MCOX2R1 (5′ GTT TTA GGT CGT TTG TTG GGA T 3′) and probes MCOX2PR1 (5′ FAM-CGT AGC TTC AGT ATC ATT GGT GCC CTA TGG T-BHQ 3′) and MCOX2P1 (5′ FAM-TTG CTC TCC CCT CTC TAC GCA TTC TA-BHQ 3′) were used in a two-step thermocycling of 95°C for 30 sec and 62° C 30 sec for 55 cycles on an iQ5 instrument (Bio-Rad, Hercules, CA). Dilutions of a plasmid containing murine COX2 sequences that was generated by PCR with the MCOX2F2 and MCOX2R1 of the murine macrophage cell line RAW264.7 (ATCC, Manassas, VI) was used as the assay standard. 0.5 ug–1 ug of prostate tissue DNA was tested using the MCOX2 primers. This assay was evaluated on PBMC DNA from 117 US blood donors and all samples tested negative, demonstrating 100% specificity of the assay. This test has a 90% and 100% sensitivity of detecting five and 10 copies of murine MCOX2 sequences in a background of 1 ug of human DNA, respectively. We were also able to detect 100–1,000 copies/cell of mtDNA sequences in the Raw264.7 mouse cell line which is equivalent to detecting a single mouse cell, assuming there are at least 1,000–10,000 mitochondria per cell.

### Phylogenetic analysis

PCR products were purified with QiaQuick PCR or gel purification kits (Qiagen, Valencia, CA) and were directly sequenced on both strands by using ABI Prism BigDye terminator kits and an ABI 3130xl sequencer (Foster City, CA). Sequences were aligned using Clustal W in the MEGA v 4.1 program [37]. Following manual editing and removal of indels, phylogenetic relationships were inferred using the neighbor joining (NJ) method implemented in MEGA v4.1 [37]. MLV phylogenies were also inferred using the program PhyML that implements the fast maximum likelihood (ML) method [38]. Support for the branching order was evaluated using 1,000 nonparametric bootstrap replicates.

### XMRV Western blot (WB)

A PMLV-infected HeLa cell line was used as antigen in the WB test as previously described in detail [21]. Briefly, PMLV-infected and -uninfected HeLa crude cell lysates were prepared as previously described [21], [39]. Plasma or serum samples were diluted 1∶50 and reacted separately to 150 ug of infected and uninfected cell lysates after protein separation through 4–12% polyacrylamide gels and then transfered to nytran membranes, as previously described [21], [39], [40]. Seroreactivity in human specimens was detected using peroxidase-conjugated protein A/G (Pierce, Rockford, IL) and chemiluminescence (Amersham, Uppsala, Sweden) [21], [39], [40]. Specimens were tested in parallel against control antigens from uninfected HeLa cells. Seroreactivity to bands in the WB blots from the infected antigens was compared to those in uninfected antigens to exclude nonspecific reactivity.

1∶500 dilutions of goat polyclonal antibodies to MLV whole virus and gp69/71Env (ATCC, Manassas, VI; VR-1537 and VR-1521, respectively) and a 1∶50 dilution of pre-immune goat sera were used as positive and negative controls, respectively, when testing of the human specimens. These two antisera have been shown to have high titers to MLV Gag (1∶32,000) and Env proteins (1∶8,000) in our WB assay, respectively [21]. In addition, using this antigen high titers to Gag and Env proteins (1∶64,000) were seen using a rabbit anti-XMRV polyclonal sera and the rat anti-spleen focus forming virus (7C10) monoclonal antibody (1∶32,000) that were previously used to detect XMRV protein expression and antibodies in prostate cancer and CFS patients, respectively [9], [18], [21]. Thus, samples were examined for seroreactivity to bands corresponding to Gag (p15, p30, pr68/80) and/or Env (gp69/71, p15E) proteins present in only the infected antigen and not the uninfected antigen. An absence of background reactivity in human samples was demonstrated previously using sera from 121 HIV and HTLV negative anonymous US blood donors collected in 1998, 13 HTLV-positive, 7 HIV-1-positive, six HIV-1/HIV-2 dual positive plasma, and pre-immune goat sera from ATCC [21].

### RNase L single nucleotide polymorphism (SNP) genotyping

DNA concentrations were first measured using the TaqMan RNase P detection kit (Applied Biosystems, Inc., Foster City, CA) following the manufacturer's instructions. 20 ng of DNA was used in the TaqMan RNase L Q462R SNP Detection Kit (C____935391_1_) using an ABI 7900HT Fast Real-time PCR instrument (Applied Biosystems, Inc., Foster City, CA) and conditions recommended by the manufacturer to determine the prevalence of wild type and mutant Q462R alleles in our study population. 20 ng of DNA in a 25 ul reaction volume was used to determine the SNP genotype. Genomic DNA from cell lines with known RNase L R462Q alleles were controls for the SNP assay (Jurkat, QQ; Raji, RR; MCF-7, RQ).
